# PROTOCOL: Online interventions for reducing hate speech and cyberhate: A systematic review

**DOI:** 10.1002/cl2.1133

**Published:** 2021-01-13

**Authors:** Steven Windisch, Susann Wiedlitzka, Ajima Olaghere

**Affiliations:** ^1^ Department of Criminal Justice Temple University Philadelphia Pennsylvania; ^2^ School of Social Sciences The University of Auckland Auckland New Zealand; ^3^ Department of Criminal Justice Temple University Philadelphia Pennsylvania

## 1 BACKGROUND

### 1.1 The problem, condition, or issue

The internet has become an everyday tool to communicate and network with people around the globe, but its perceived anonymity, availability, and instant access have made it an environment conducive to spreading hateful content and connecting to like‐minded individuals with similar hateful ideologies. Hate speech and other prejudice‐motivated behavior, however, need to be considered on a continuum of victimization, and “like other social processes, [be seen as] dynamic and in a state of constant movement and change, rather than static and fixed” (Bowling, 1993, p. 238). It is a social process that is marked by multiple, repeat, and constant victimization (Bowling, 1993), with victims no longer distinguishing between specific hateful events, and rather normalizing experiences of hateful conduct “as an everyday, unwanted but routine reality of being 'different'” (Chakraborti, 2016, p. 581). Understanding hateful behavior and victimization as a process allows us to connect “low‐level” incidents of hateful behavior to the more serious and life‐threatening incidents at the more extreme end of the spectrum (Bowling & Phillips, 2002). The Christchurch attacks in New Zealand and their link to hateful communication on the online platform 8chan is only one such example of how online hate speech and cyberhate can escalate to “in real life” attacks, leaving the online sphere and spilling into the offline world. As per Allport's (1954) *scale of prejudice*, more extreme forms of prejudice‐motivated violence are founded on “lower level” acts of prejudice and bias, therefore, hateful content online should not be ignored. Intervening online to interrupt or counter hateful behavior already at the lower end of the scale of prejudice becomes important; online interventions which are to be identified and synthesized through this systematic review.

Allport's (1954) scale of prejudice will be the basis for this systematic review. Early on, Allport (1954) asserted that individuals with negative attitudes toward groups are likely to act out on these prejudices “somehow, somewhere” (p. 14), and that the more intense such negative attitudes are, the more hostile the action will be. Allport (1954) put forward a scale of acts of prejudice to illustrate different degrees of acting out negative attitudes, a scale that starts with *antilocution* (or what we call hate speech), described as explicitly expressing prejudices through negative verbal remarks to either friends or strangers (Allport, 1954). *Avoidance* is the next level on the scale of prejudice, with people avoiding members of certain groups, followed by *discrimination*, where distinctions are made between people based on prejudices, which leads to the active exclusion of members from certain groups (Allport, 1954). This level of acting on prejudices is routed in institutional or systemic prejudices, for example, in the differential treatment of people within employment or education practices, but also within the criminal justice system, or through social exclusion of certain minority group members. *Physical attack* is the next level on the scale of prejudice, which includes violence against members of certain groups by physically acting on negative attitudes or prejudices. The last level is *extermination*, which is the ultimate act of violence against members of specific groups, an expression of prejudice that systematically eradicates an entire group of people (e.g., genocide or lynchings; Allport, 1954). Allport's (1954) scale of prejudice makes it clear how hate speech/cyberhate is connected to more extreme forms of violence motivated by specific prejudices and biases, with hate speech (or antilocutions) being only the starting point on a 5‐point continuum (Bilewicz & Soral, 2020). The importance of this scale of prejudice is not only that it clearly illustrates a range of different ways and intensity levels to act out prejudices, but also the “progression from verbal aggression to physical violence or, in other words, the performative potential of hate speech” (Allport, 1954; Kopytowska & Baider, 2017, p. 138). This is where interventions at the lower level of the scale of prejudices, interventions targeting hate speech/cyberhate, become important.

There is no universal definition of hateful conduct online, but there is some consensus that hate speech targets disadvantaged social groups (Jacobs & Potter, 1998). Bakalis (2018) more narrowly defines cyberhate as “any use of technology to express hatred towards a person or persons because of a protected characteristic—namely race, religion, gender, sexual orientation, disability and transgender identity” (p. 87). Another definition that also points out the ambiguity and challenges involved with identifying more subtle forms of hate speech, and also making reference to the potential threat of hate speech escalating to offline violence, is that put forward by Fortuna and Nunes (2018), who analyzed various definitions of hate speech “Hate speech is language that attacks or diminishes, that incites violence or hate against groups, based on specific characteristics such as physical appearance, religion, descent, national or ethnic origin, sexual orientation, gender identity or other, and it can occur with different linguistic styles, even in subtle forms or when humour is used” (p. 5). In this systematic review, we also distinguish hate speech/cyberhate specifically from other forms of harmful online activity, such as cyber‐bullying, harassment, trolling or flaming, as perpetrators of such online behavior repeatedly and systematically target specific individuals to cause upset, to seek out negative reactions, or to create discord on the internet. In contrast, hate speech/cyberhate is more general and does not necessarily target a specific individual (Al‐Hassan & Al‐Dossari, 2019), instead hate speech/cyberhate heavily features prejudice, bias and intolerance toward certain groups within society. With the majority of hate speech happening online, interventions that take place online are an important way to challenge prejudice and bias, potentially reaching masses of people across the globe.

The unique feature of the internet is that such individual negative attitudes toward minority groups and more extreme hateful ideology can find its way onto certain platforms and can instantly connect people sharing similar prejudices. By closing the social and spatial distance, the internet creates a form of *collective identity* (Perry, 2000, p. 123) and can convince individuals with even the most extreme ideologies that others out there share their views (Gerstenfeld et al., 2003). In addition, the enormous frequency of hate speech/cyberhate within online environments creates a *sense of normativity to hatred* and the potential for acts of intergroup violence or political radicalization (Bilewicz & Soral, 2020, p. 9). It is, therefore, important to challenge this *hate speech epidemic* (Bilewicz & Soral, 2020), especially since hate movements have increasingly crossed into the mainstream (Perry, 2000). With hate speech/cyberhate posing a threat to the social order by violating social norms (Soral et al., 2018), perceptions of social norms as either supporting or opposing prejudice has been found to have an influence on how individuals react online (Hsueh et al., 2015). Seeing other people post prejudiced (opposed to antiprejudiced) comments online can lead to the adoption of an online group's biases and can influence an individual's own perceptions and feelings toward the targeted stigmatized group (Hsueh et al., 2015). In addition, research around desensitization also suggests that being exposed to hate speech leads to desensitization, which further leads to an increase in outgroup prejudice toward groups targeted by such speech (Soral et al., 2018). With society increasingly recognizing that it is inappropriate to express prejudices in public settings, many interventions will include some form of social norms nudging to reduce such prejudices; interventions that “nudge behavior in the desired direction” (Titley et al., 2014, p. 60). Therefore, hate speech not only affects minority group members, but also has an influence on opinions of majority group members (Soral et al., 2018), which makes strategies that can elicit change in people's prejudice‐related attitudes crucial (see, e.g., Zitek & Hebl, 2007).

Governments around the world face increased demand for understanding and countering hateful ideology and violent extremism both online and offline (e.g., the Christchurch Call in New Zealand). The U.S. Government's 2011 CVE Strategy highlights the importance of ongoing research and analysis, the sharing of knowledge and best practices internationally, and the countering of hateful ideologies and propaganda (see also Department of Homeland Security, 2016, 2019). The goal of this systematic review is to use an integrated and interdisciplinary approach to examine the effectiveness of online campaigns and strategies for reducing hate speech and cyberhate.

### 1.2 The intervention

The internet also provides an opportunity to reach masses of people who have been exposed to hateful content and ideology online, therefore, this systematic review will focus on online interventions addressing online hate speech and cyberhate. The specific settings where we would expect to see the online interventions deployed will be on websites, text messaging applications, and online and social media platforms including, but not limited to, Facebook, Instagram, TikTok, WhatsApp, Google, YouTube, and Snapchat. As mentioned previously, many online interventions will be based on social norm nudges to reduce online hate. These interventions aim to change people's online behavior and encourage individuals or groups to conform to established social norms. The communication of social norms can happen through establishing community standards on online platforms themselves (e.g., Facebook, Twitter, etc.), through more formal online training courses, or through anti‐hate speech/anti‐cyberhate campaigns teaching people to recognize hate, embrace diversity, and stand up to bias. Such prevention campaigns are designed to challenge bias and build ally behaviors by supplying people with constructive responses to combat, for example, antisemitism racism, and homophobia, as well as provide resources to help people explore and critically reflect on current events. Other interventions may add messages to hateful online comments, counter hateful content or extremist ideology, or redirect people to more credible sources.

### 1.3 How the intervention might work

Both peers and parents have been found to foster racial consciousness and identity development, define interracial relationships and cultivate ethnic heritage and culture (Hagerman, 2016). Socialization influences how children understand their group's social position and their membership within that group by providing an understanding of racial, religious, and sexual privilege (Bowman & Howard, 1985). Socialization often reflects peers' and parents' experiences with racism, discrimination, and their ideological perspectives about race, religion, or sexuality (Umaña‐Taylor & Fine, 2004). This is important because peers and parents who feel discriminated against or believe that the “other” is a threat may impart their prejudices to their children or friends, which could lead them to interpret the social world with similar discriminatory views and/or behavior. Individuals who feel socially alienated or rejected are especially vulnerable to such socialization practices as they feel that adopting these views will provide them with a sense of acceptance and belonging (Leiken, 2012).

Regardless of how an individual develops certain racial, religious, or sexual biases, the online interventions under review are expected to target and reduce the production of original hateful content such as antisemitic Tweets and/or homophobic blog posts as well as the consumption of hate speech material (e.g., watching or reading hate speech videos or blogs). For example, some interventions take a rather broad messaging approach by implementing racial sensitivity and diversity training through Public Service Announcements, peer‐to‐peer dialogue workshops, or films that provide opportunities for youth and adults to self‐reflect and learn about historical oppression, people of color, women, and the LGBTQIA+ community from credible sources. The factual understanding of diverse groups is often supplemented by experiences with people within the group. These educational programs often identify a cultural guide who is willing to introduce youth to new experiences and who can aid in processing thoughts, feelings, and behaviors. These interventions intend to dispute and contradict negative stereotypes associated with specific cultures, people, and institutions by sharing different points of view based on human rights values such as openness, respect for difference, freedom, and equality (Gomes, 2017). Moreover, such interventions tend to involve blanket bans on specific behaviors enforced through the public promotion of norms or individual sanctions enforced by moderators.

Other interventions, such as the “Redirect Method,” are narrower in their messaging. These interventions generate curated playlists and collections of authentic content that challenge hate speech/cyberhate narratives and propaganda (Helmus & Klein, 2018). For instance, people who are directly searching for extremist content online may be linked to videos and written content that confronts such claims. These videos are designed to be objective in appearance instead of containing material that explicitly counters extremist propaganda. The underlying goal of this type of interventions is to provide credible content that effectively undermines extremist messaging but does not overtly attack the source of propaganda. In addition to confronting hate speech narratives, these interventions provide users with links to numerous social services such as anger management training, drug and alcohol treatment, and mental health resources. Online platforms, such as Twitter and Facebook, have also started to employ a similar method, redirecting people who comment on or share “fake news” or conspiracy theories, which often are fraught with prejudicial undertones and are harmful to minority groups, to more credible content and news sources.

The aforementioned interventions are designed to counter‐balance these biased perceptions (e.g., unsupported claims of the Black community as criminal or the LGBTQIA+ community as pathologized) Blacks as criminals, LGBTQIA+ as pathologized) by blunting the occurrence of racist discourse and reducing the likelihood these individuals will internalize and normalize racial, religious, and/or sexual prejudices (Qian et al., 2019). Being in new situations is uncomfortable and often awakens fears and apprehensions that can block our experiential development. Acquiring information or being exposed to minority‐run businesses, poverty, and writings from minority authors allows a person to understand the thoughts, hopes, fears, and aspirations of the people outside their racial perspective rather than from the perspective of the majority society (Dunham et al., 2013; Lee et al., 2017). Doing so, counters racist programming by challenging hegemonic beliefs, which can lead to the acceptance of tolerant attitudes and the reduction of hateful expressions online.

### 1.4 Why it is important to do this review

Findings from the proposed review will enhance our understanding of the effectiveness of online anti‐hate speech/anti‐hate interventions, will help ensure that programming funds are dedicated to the most‐effective efforts, and will play a critical role in helping individual programs improve the quality of service provisions. It will inform governments and policymakers of the current state of such online efforts, what works and which modes of interventions to implement, and help guide economically viable investments in nation‐state security.

### 1.5 Prior reviews

Our search of the scholarly literature identified one review, Blaya (2019), as similar to the proposed topic. Blaya's (2019) review, however, focused on the prevalence, type, and characteristics of existing interventions for counteracting cyberhate and did not include a meta‐analysis. Two other similar reviews focused on exposure to extremist online content (Hassan et al., 2018) and communication channels associated with cyber‐racism (Bliuc et al., 2018). A search of the Campbell Library using key terms (hate OR radical*) returned two protocols and one review identified for further inspection to assess potential overlap. The protocols include “Psychosocial processes and intervention strategies behind Islamist deradicalization: A scoping review” by de Carvalho et al. (2019) and “Police programs that seek to increase community connectedness for reducing violent extremism behavior, attitudes and beliefs” by Mazerolle et al. (2020). A further review on a similar topic is a recently completed Campbell review (January 2020), “Counter‐narratives for the prevention of violent radicalization: A systematic review of targeted interventions” by Carthy et al. (2018) at the National University of Ireland, Galway.

Our proposed review is distinguished from the de Carvalho et al. (2019) review in that we are focusing on hate speech and cyberhate generally without delimiting our approach to a specific type of radicalization (e.g., Islamist). Furthermore, we are electing to complete a systematic review and meta‐analysis. Likewise, the protocol by Mazerolle et al. (2020) focuses on interventions involving police officers either as initiators, recipients, or implementers of community connectedness interventions. Our review will focus specifically on any online intervention, which may or may not involve police, but police will not be the focus nor be the basis of the online intervention strategy. Judging from Carthy et al. (2018) protocol, we anticipate our review will also capture counter‐narrative interventions, but will differ based on setting, timing, and scope of interventions. Specifically, we are interested in online interventions that extend beyond counter‐messaging campaigns to include a broad array of interventions outlined above and extend beyond radicalization to include everyday hate and prejudice. In addition to conducting a meta‐analysis, the proposed review would build on Blaya's (2019) work by expanding the population parameters to include both adolescents as well as adults. Blaya (2019) limited her search to include interventions aimed toward youth, young people, children, young adults, adolescents, children, and teenagers and did not focus on extremism.

## 2 OBJECTIVES

The main objective of this review is to synthesize the available evidence on the effectiveness of online interventions aimed at reducing the creation and/or consumption of online hate speech/cyberhate material.

The specific research questions guiding this review include:

1. *To what extent are online interventions effective in reducing online hate speech/cyberhate?*
2. *How is effectiveness related to the type of online hate speech/cyberhate intervention used?*
3. *How is effectiveness related to the characteristics of individuals experiencing the online hate speech/cyberhate intervention (e.g., age, gender, race/ethnicity, offense history, childhood trauma)?*

## 3 METHODS

### 3.1 Inclusion criteria

#### 3.1.1 Types of study designs

Both experimental and quasi‐experimental quantitative studies will be included. These study designs will address research questions #1 to #3. Eligible quantitative study designs include the following:

##### 3.1.1.1 Experimental designs

Eligible experimental designs must involve random assignment of participants to distinct treatment and control group(s). Designs that involve quasi‐random assignment of participants such as alternate case assignment are also eligible and will be coded as experimental designs.

##### 3.1.1.2 Quasi‐experimental designs

All eligible quasi‐experimental designs must include a comparison group of participants compared to participants in the treatment condition. Eligible studies include those that report matching procedures (individual‐ or group‐level) and statistical procedures employed to achieve equivalency between groups. Statistical procedures may include, but are not limited to, propensity score matching, regression analysis, and analysis‐of‐covariance. Furthermore, in anticipation of a limited quantitative evidence base, we will also include quasi‐experimental studies with unmatched comparison groups that provide baseline assessment of outcomes for both groups. Finally, time‐series analyses will also be included. Eligible time‐series design include short‐interrupted time series designs with a control group (<25 pre/post observations) and long‐interrupted time series designs with or without a control group (more than 25 pre/post observations). Ineligible quasi‐experimental designs include studies that utilize a comparison group consisting of participants who either refused to participate in the study or who initially participated in a study, but then dropped out prior to the start of a study.

#### 3.1.2 Nature of eligible comparison conditions

Eligible comparison conditions include other online interventions or conditions in which participants do not receive or experience an online intervention.

#### 3.1.3 Types of participants

Both youth and adult participants of any racial/ethnic background, religious affiliation, gender identity, sexual orientation, nationality, or citizenship status will be eligible for this review. The eligible youth population will be study participants with a minimum age of 10 through age 17. The eligible adult population will be study participants with a minimum age of 18 and older.

Studies in which only a subset of the sample is eligible for inclusion—for example, if a study subject participates in both online and offline hate speech interventions—will be excluded. We do not anticipate excluding studies based on sample eligibility, as our inclusion criteria will be wide‐ranging, and we will take reasonable steps to locate studies that only involves online interventions. We will resolve differences of opinion regarding the eligibility of a study for inclusion through discussion and consensus. If agreement cannot be reached, we will elicit the opinion of a subject matter expert, whereby the final list of included and excluded studies will be decided. Since these studies will be excluded, they will be unavailable and cannot be calculated in the meta‐analysis and any related subgroup/sensitivity analysis.

#### 3.1.4 Types of interventions

We adopt Blaya's (2019) four‐part typology of intervention strategies to outline the potential universe of eligible interventions. The first intervention strategy is the adaptation of legal responses to hate speech/cyberhate, which includes the countering of violent extremism and aims to address cybercrime. More specifically, online interventions that are eligible range from disrupting hateful content online via specific “crackdowns” (e.g., server shutdowns, deletion of social media accounts) to responding to online hate using targeted strategies (e.g., through counter‐narratives, modifying hateful content). Examples of previous studies focusing on online crackdowns include the monitoring and investigation of online accounts and content takedowns, online content monitoring and censorship (Alvarez‐Benjumea & Winter, 2018), modifying hateful online comments to nonhateful comments (Salminen et al., 2018), and possibly changing algorithms to divert users out of online echo chambers. We are also interested in interventions such as the recent take‐down of 8chan after this online platform was linked to “in real life” attacks in New Zealand and the United States, and if interventions exist that disrupt further hateful online content and radicalization after similar trigger events.

Disrupting hateful content online via such crackdowns has brought up free speech concerns, as well as concerns around online users and hateful groups just moving on to other online platforms. Responding to hateful content online using targeted strategies has, therefore, been suggested as an effective online intervention. Examples include message priming using the endorsement from religious elites (Siegel & Badaan, 2020), the use of bots to sanction online harassers (Munger, 2017), automatically generating responses to intervene in online conversations where hate speech has been detected (Qian et al., 2019), and redirecting online users to YouTube videos debunking, for example, ISIS recruiting themes (https://redirectmethod.org/). Our systematic review will include a broader range of online interventions, many of which have only recently emerged.

Two other strategies identified by Blaya (2019) are the automatic identification and regulation of hate speech/cyberhate using technology as well as the creation of online counter‐spaces and counter‐communication initiatives. These interventions include online counter‐narrative marketing campaigns, the establishment and/or use of online counter spaces, online education‐based interventions, online citizenship training, and online legislative initiatives narrowly defined to address extremist ideologies and hate speech that incites targeted violence and radicalization. In general, such interventions seek to prevent or minimize the occurrence of violent extremism or radicalization, including the spread of hate speech and extremist propaganda, by disrupting recruitment channels and creating opportunities to leave such groups.

The fourth and final intervention strategy eligible for this systematic review involves educational programs that, for example, provide people with online literacy skills and challenge racism (Blaya, 2019). We will include online empowerment/resilience approaches, policy programs with an online component (e.g., Prevent and Exit programs), and educational and awareness‐raising online interventions. Some of these interventions may evaluate behavioral changes by individuals no longer engaging in the creation and/or consumption of cyberhate and extremist material online. These online interventions may be sponsored by nonprofit and nongovernmental organizations, internet service providers, or policy or governmental agencies in the case of legislative interventions. The comparison condition may be routine exposure and engagement to hate speech/cyberhate or another online intervention.

#### 3.1.5 Types of outcome measures

The primary outcome of interest is the creation and/or consumption of hateful content online. By creation, we refer to the production and authorship of original hateful content such as posting antisemitic Tweets, uploading racist YouTube videos, and/or writing homophobic blog posts. The consumption of hate speech material may include visiting or being a member of a hate website/online group, watching or reading hate speech videos or blogs, being a target of online hate speech/cyberhate, or reporting hate speech material.

Secondary outcomes of interest include affective and emotional states of study participants such as anger, fear, emotional unrest, depression, anxiety, mood swings, and attitudes toward hate speech/cyberhate. Eligible studies must report a primary or secondary outcome (or both) to be included.

There will be no exclusion criteria on the source of outcome data. Data for the primary and secondary outcome measures can be obtained from any courses including institutional records, direct observations, surveys or questionnaires completed by participants.

##### 3.1.5.1 Adverse effects

We will include any measure of unintended adverse effects from strategies to increase the scale of implementation of potentially effective anti‐hate speech and deradicalization interventions for participants. These could include adverse changes to emotional or psychological well‐being, defensiveness, guilt, shame, resistance to the teaching, miscommunication, creation of barriers, and dysfunctional adaptation behaviors. Adverse effects can also include nonindividual effects such as a relocation of hate speech/cyberhate to other platforms instead of a reduction of hate speech/cyberhate. All adverse effects described in eligible studies will be included in the synthesis.

#### 3.1.6 Other inclusion criteria

We will focus on the period between 1990 and the current year, 2020. The period restriction starting with the year 1990 considers when the internet transitioned to a wider infrastructure and broad‐based global community (Leiner et al., 2009). We are opting for an inclusive approach in bounding the lower end of our search period to 1990. While the odds may be slim, it is conceivable hate speech/cyberhate was present online through mailing lists or emails and some studies may capture this. Our population of studies will also be limited to studies published in English, German, Persian, and Arabic, but inclusive of studies completed in any geographical region, as we are focused on online content that can be consumed and shared across geographic and nation‐state boundaries. The language parameters reflect the language abilities of the review team. Our full‐text coding will consider where studies were conducted and, if possible, the geographic location of included study participants.

Any changes in eligibility criteria will be agreed prospectively between the members of the review team. These will be documented and reported as a discrepancy from the protocol in the review. In the advent of a change in eligibility, we will rescreen citations.

### 3.2 Search strategy

We will use Zotero to manage references and implement the search strategy below and will document the search process using the following fields: date, reviewer initials, database/website/journal searched, final search string, total yield, and notes to capture any aberrant cases. Search terms will be developed based on terminology representative of implementation and dissemination research and include search filters used in previous reviews (Blaya, 2019). The search strategy will be conducted by using the search terms specified below within the search fields of Title, Abstract, Keywords (supplied by the author), and indexing terms. We will also use an automated screening feature in DistillerSR for title and abstract screening and track excluded titles at this phase.

1. Setting search terms:
  online OR “social media” OR internet OR Twitter OR Facebook OR 8chan OR 8Kun OR Gab OR Telegram OR TikTok OR Reddit OR WhatsApp OR Instagram OR “social networking site*” OR “cybervictimization” OR “online incivility”
  AND
2. Extremism/radicalization/hate terms:
  “hate speech” OR cyberhate OR extrem* OR narrative OR racis* OR radical* OR speech OR ideolog* OR islamophobi* OR homophobi* OR transphobi* OR misogyny OR disablism OR discrim* OR terror*
  AND
3. Treatment terms:
  interven* OR option* OR strategy* OR “counter narrative*” OR “nudge” OR “norm* intervention” OR “norm* nudge” OR counternarrative* OR “alternative narrative*” OR campaign* OR counter* OR peer‐to‐peer OR prevent* OR disrupt* OR stop* OR fight* OR redirect* OR censoring hate content”
  AND
4. Evaluation terms:
  comparison* OR quantitative OR quasi‐experiment* OR survey* OR interview* OR poll* OR mixed‐methods OR individual‐level OR group‐level OR control* OR experiment* OR study OR studies OR evaluat* OR MTurk OR longitudinal OR random* OR “digital method*” OR “machine learning” OR “natural language processing” OR multisectoral OR review*
  AND
5. Year limiter:
  1990–2020

#### 3.2.1 Electronic sources

The search strategy described above will be applied to the following databases, which cover easily accessible sources as well as gray literature. Gray literature includes reports, working papers, white papers, government documents, and generally non‐peer reviewed works.

##### 3.2.1.1 Databases

###### 3.2.1.1.1 Databases from major platforms

**Table:** 

EBSCOHost Research Database	
Academic Search Complete	Education Resources Information Center (ERIC)
Communication and Mass Media Complete	Military and Government Collection
Communication Abstracts	PsycARTICLES
Criminal Justice Abstracts with Full Text	Psychology and Behavioral Sciences Collection
EBSCOhost	PsycINFO

**Table:** 

ProQuest	
Applied Social Sciences Index & Abstracts (ASSIA)	ProQuest Criminal Justice
Criminal Justice Abstracts with Full Text	ProQuest Dissertation & Theses Global
Education Resources Information Center (ERIC)	ProQuest Political Science Database
Gender Watch	ProQuest Social Science Journals
International Bibliography of the Social Sciences (IBSS)	ProQuest Sociology
National Criminal Justice Reference Service (NCJRS)	Sociological Abstracts
Public Affairs Information Service (PAIS)	Worldwide Political Science Abstracts
Policy File Index	

**Table:** 

Sage
Sage Journals Online
Sociology (Sage Full‐Text Journal Collection)

**Table:** 

Databases—Individually searched	
Academic One File	International League Against Racism and Anti‐Semitism (LICRA)
AFPD—Australian Federal Police Digest	Irish Network Against Racism
ArticleFirst	JSTOR
Cambridge Journals Online	LLMC Digital
CINCH: Australian Criminology Database	Multicultural Australia and Immigration Studies—Aboriginal and Torres Strait Islander Subset (MAIS‐ATSIS)
Columbia International Affairs Online (CIAO)	Journals@Ovid
Council of Europe	Oxford Journals Online
Declassified Documents Reference System	Oxford Scholarship Online
European Commission	Project Muse
Global Issues in Context	PsychiatryOnline
Google Scholar	Sage Knowledge ebook collection
Don M. Gottfredson Library of Criminal Justice Gray Literature Database	ScienceDirect
Fundamental Rights Agency	Scopus
Govinfo	Social Science Research Network
HeinOnline (All databases)	Social Sciences Citation Index
Homeland Security Digital Library (HSDL)	SpringerLink
Human Right League	Taylor & Francis Online
Index New Zealand: INNZ	Web of Science (All databases)
Ingenta Connect	Wiley Online Library
International Federation of Human Rights	WorldCat

**Table:** 

Journals	
Annual Review of Sociology	Journal of Hate Studies
Annual Review of Criminology	Journal for Deradicalization
Behavioral Sciences of Terrorism and Political Aggression	Journal of Policing, Intelligence and Counter Terrorism
Critical Studies on Terrorism	Perspectives on Terrorism
Dynamics of Asymmetric Conflict	Studies in Conflict & Terrorism
Intelligence and National Security	Terrorism and Political Violence

**Table:** 

Websites	
ADL Combating Hate—CYBERHATE	MANDOLA—Monitoring and Detecting OnLine Hate Speech
BRICkS—Building Respect on the Internet by Combating Hate Speech	Ministry of Justice (UK, New Zealand), Department of Justice in each Australian state or territory
Counter Narrative Handbook	RAND
	RAND Europe
Don M. Gottfredson Library of Criminal Justice Gray Literature Database	Stand Up to Hate
eMORE Project—Monitoring and Reporting Online Hate Speech in Europe	The Alan Turing Institute Online Hate Research Hub
European Commission against Racism and Intolerance (ECRI)—On combating hate speech	The Online Hate Prevention Institute
Hate Speech Watch	they can't—Fighting Antisemitism & Terrorism Online
Home Office	Together against Hate on the Net
INACH—International Network Against Cyber Hate	UNESCO—Countering Online Hate Speech
INHOPE	United Nations—General recommendation No. 35 (Combating racist hate speech)
International Network for Hate Studies online library	Urban Institute
iSCA—Institute for the Student of Contemporary Antisemitism at Indiana University Bloomington	VOX‐Pol
Light on Project	YouTube Creators for Change

##### 3.2.1.2 Additional searches

We will also complete forward citation searching and backward searches, or reference harvesting, of relevant reviews we come across in our search in addition to prior reviews and reports (e.g., Blaya, 2019; Bliuc et al., 2018; Brown & Cowls, 2015; Hassan et al., 2018; Strachan, 2014) and will search the reference lists of included studies eligible from full‐text screening. We will complete forward and backward searches for any article that comes from the *Journal for Deradicalization* and the *Journal of Hate Studies* as the content from these two journals closely align with the review topic. Additionally, we will contact study authors and journal editors (i.e., *Dynamics of Asymmetric Conflict*, *Journal for Deradicalization*) to capture additional studies that may be in press.

We will document all steps of the search process in sufficient detail to ensure future replicability and correct reporting. This will include a PRISMA flowchart, registration of excluded studies and dates at which the search was conducted. If the initial search date is more than 12 months from the intended publication date for the review, we will rerun searches and fully incorporate new eligible studies. We will record the following information for each conducted: the date of search, database and platform searched, search syntax, any modifications or restrictions to the search, and the *N* for the search exported. When forward searching is completed, we will use Google Scholar because the database will identify both published and unpublished literature.

### 3.3 Data collection

#### 3.3.1 Description of methods used in primary research

The anticipated methods that prior studies employ range from experimental designs to assessment and impact evaluations. Previous research has relied on experimental designs to test treatments on discussions of current social topics (Alvarez‐Benjumea & Winter, 2018), message priming to reduce sectarian hate speech online (Siegel & Badaan, 2020), as well as randomized field experiments on Twitter using bots to sanction online harassers (Munger, 2017). Similarly, machine learning and keyword matching has been used to distinguish between hateful and nonhateful comments on social media platforms. Finally, using neural network modeling to modify hateful online comments to nonhateful comments, researchers have been able to evaluate results manually as well as through crowd experiments and statistical tests (Salminen et al., 2018).

#### 3.3.2 Criteria for determining independent findings

The primary analysis for the effect of online interventions on content creation and consumption of hate speech/cyberhate or extremist and non‐extremist material will rely on self‐report measures and at any time point post‐intervention (e.g., 3, 9, 12 months, etc.). Secondary analyses will explore whether intervention effects on affective and emotional states differ across different self‐report measures of exposure or engagement (e.g., visiting, posting, online membership, or reporting/flagging). These analyses will handle the issue of statistical dependencies by using meta‐regression with the robust variance estimator in Stata to implement robust variance estimation developed by Hedges et al. (2010).

We anticipate three issues relating to the determination of independent findings that will need to be addressed in this review. First, documents may report on multiple studies and/or multiple outcomes. Our protocol for this situation will be to allow documents to contribute multiple effect sizes, but only contribute one effect size for each outcome. If a document provides multiple effect sizes for an outcome, we will model the statistical dependencies using robust variance estimation as noted above. The second issue of independence is where multiple documents report data from the same evaluation. A research study will be treated as unique only if the study sample does not include study participants involved in any other coded study. Studies or reports generated from the same sample will be coded as a single study. In these cases, the study with the most complete information will be the primary study and the related references will be cross‐referenced with a related study ID. We will treat dependent studies as a single study and use all sources to calculate effect sizes for each outcome. Third, for studies that report outcome data at multiple time points, we will perform separate analyses: short‐term (3–6 months), medium‐term (7–9 months), and long‐term follow‐up (10–12 months or longer).

#### 3.3.3 Selection of studies

After the removal of duplicates, the abstract and titles will be single screened via DistillerSR and screeners will be asked to assess the eligibility of each of the studies via the following questions:

a. Is the study in English, German, Persian, or Arabic? Yes/No/Unknown
b. Was the study conducted between 1990 and 2020? Yes/No/Unknown
c. Does the study mention an intervention/prevention? Yes/No/Unknown
d. Does the study include an online component? Yes/No/Unknown
e. Does the study address hate speech/cyberhate? Yes/No/Unknown

Based on the above preliminary questions, screeners will then assess the following question: Does the study address an online prevention or intervention with an impact on online hate speech/cyberhate or radicalization? Yes/No/Unsure

Any title and abstracts where screeners have indicated “yes” to the screener question will be pushed through to full‐text screening. If screeners were unsure of the eligibility of the study, these title and abstracts will be double‐screened. If these remain unsure due to limited information within the title and abstract, these studies will also be pushed through to full‐text screening.

DistillersSR's “continuous AI reprioritization” feature will learn from abstracts that screeners have accepted or rejected and will present the abstracts likely to be included in the systematic review first, which allows for a speedier abstract and title screening process. Once studies are deemed potentially eligible at the title and abstract screening phase, DistillerSR will be used for full‐text screening via data collection forms. Similar to the title and abstract screening phase, the full texts will be single‐screened unless double‐screening is necessary if questions remain about the eligibility of the full text studies, then the opinion of a second reviewer will be sought.

#### 3.3.4 Data extraction and management

Two review authors, also unblinded to author or journal information, will independently extract information from the included studies. This information will be recorded in a data‐extraction form that will be piloted before initiation of the review. Discrepancies between reviewers regarding data extraction will be resolved by consensus or if required via a third reviewer. Data collection forms will be created and hosted online using DistillerSR (see Appendices A and B for specific coding forms). Basic information about included studies will be described as a narrative and included in a study characteristics table. Specifically, we will describe and tabulate information consistent with MECCIR reporting standards (i.e., R61–R70) including sample size, methods/study design, setting/context, participants, interventions/comparison characteristics, outcome characteristics, effect size data, dates, funding sources, and declarations of interest. As part of data extraction, we will check the accuracy of all numeric data in the review. Where information is unavailable from published reports, we will contact study authors to obtain such data.

#### 3.3.5 Assessment of methodological quality/risk of bias

Methodological quality and risk of bias will be coded as data is extracted for study, intervention/comparison, and outcome characteristics. We will evaluate risk of bias using the Cochrane Collaboration's risk of bias tools. In particular, for randomized quantitative studies (RoB 2), we will focus the risk of bias assessment on select domains including bias arising from the randomization process and bias in measurement of the outcome (Sterne et al., 2019). For nonrandomized quantitative studies (ROBINS‐I), we will focus the risk of bias assessment on bias in the selection of participants and all domain of bias in postintervention (Higgins et al., 2011; Sterne et al., 2016). At the study level, we will code for the type of experimental and quasi‐experimental design based on assignment (e.g., matching, wait list control, cohort, etc.). Our ratings for evaluating risk of bias will be “low risk,” “some concerns,” and “high risk” of bias. In accordance with MECCIR R72, ratings will be presented in a risk of bias/study quality table for each included study. Furthermore, the replicability of included studies may be a problem, as platforms or users may delete hateful content. This may lead to publication bias or p‐hacking. At the end of the protocol, we address how we assess publication selection bias. For the latter issue, we use the bias in the selection of the report result domain in RoB 2 and ROBINS‐I to evaluate risk of bias related to p‐hacking.

#### 3.3.6 Dealing with missing data

Missing data may be in the form of missing studies, missing outcomes, missing summary data, or missing participants. We do not anticipate missing studies, as our search strategy will be comprehensive, and we will take all reasonable steps to locate the full texts of eligible studies. Following the recommendation of Pigott and Polanin (2020) we will handle missing outcomes, missing summary data and missing participants by first contacting study authors via email with a request to provide the missing information and second, by making reasonable and appropriate inferences based on a study's population and setting, if feasible. If data are not available, we will not impute values. Rather, we will either implement a complete case analysis or maximum likelihood estimation depending on the final number of studies included. We will report the extent of missing data within individual studies in the “Risk of bias” tables.

#### 3.3.7 Studies with two or more treatment groups

Procedures described in the Cochrane Handbook for Systematic Reviews of Interventions will be followed for trials with more than two intervention or comparison arms to avoid double counting of study participants in the meta‐analysis (Higgins & Green, 2011). Specifically, where possible, active intervention arms will be combined and compared against usual care or control conditions. If this is not possible, a single pair of intervention—control conditions will be selected for comparison. The selection of such a comparison will be undertaken by review pairs who will be blind to results describing intervention effects.

### 3.4 Analysis

#### 3.4.1 Planned synthesis of results

The primary outcome for this review is content creation and consumption of hate speech/cyberhate, extremist material, and nonextremist online material. We anticipate the underlying nature of data for this outcome will be continuous. As such, the effect size of choice for this review will be the standardized mean difference. In the case of quasi‐experimental designs with statistical adjustments for baseline differences, the regression coefficient from a logistic regression model will be coded as the logged odds ratio along with the reported standard error. Where studies report dichotomous outcomes, we will use the logit method for transformation and divide the logged odds ratio by 1.83 to make it comparable to the standardized mean difference effect size (Lipsey & Wilson, 2001). The meta‐analysis will be conducted using a random effects model with robust variance estimation for estimating the mean effect size and its confidence interval. The meta‐analysis will be performed using Stata IC/16 and specifically the *robumeta* macro to use robust variance estimation (Hedberg, 2014). Furthermore, given our interest in the timing of interventions and subsequent effects, where possible we will meta‐analyze results by posttreatment periods (e.g., 3, 6, and 9 months, etc.). We will also adjust for baseline outcome data by including these measurements as covariates in a regression model (Deeks et al., 2020; also see Fu et al., 2013).

If studies using different scales are combined, we will ensure that higher scores for continuous outcomes all have the same meaning for any particular outcome. Specifically, we will explain the direction of interpretation and report when reversing scores to align direction is done. Finally, we will check continuous outcome measures for skewness and, if substantial departures from normality are observed, we will transform these data prior to meta‐analysis. If we are unsuccessful at transforming the data, we will attempt to contact the author of the study and request additional data.

#### 3.4.2 Subgroup analysis and assessment of heterogeneity

We will split included studies into subgroups based on study design, demographics (e.g., political affiliation, age, etc.), and intervention characteristics to explain homogeneity. Heterogeneity will be measured using *I* ^2^ in conjunction with *τ* ^2^ and *χ* ^2^.

Given that a small number of eligible studies are expected for this review, we do not anticipate conducting moderator analyses beyond differences in study design, and it is highly unlikely that we will have a sufficient number of studies to conduct a meta‐regression. As such, we will group studies according to study design. Specifically, whether studies are randomized controlled trials (RCTs) or non‐RCTs, we will estimate variance components for within‐studies groups (use fixed effect models if variance components are the same, random effects models if different) and test whether the mean effect size from the RCT‐only group differs from the mean effect size from the non‐RCTs group.

#### 3.4.3 Planned moderator analyses

To explore heterogeneity among effect sizes, we will use the analog‐to‐the‐analysis of variance method for a single categorical variable to perform moderator analyses. For continuous moderators or multiple moderators, we will use meta‐analytic regression. All moderator analyses will be performed under a random effects model.

### 3.5 Confirmatory analyses

We assume the following factors may have an impact on the effect size and will inform our a priori moderator analyses: the quality of the study design (e.g., experiment, quasi‐experiment) and the focus of the intervention (e.g., anti‐racism, antisemitism general racism, xenophobia, homophobia, etc.) (objective 2).

### 3.6 Exploratory analyses

*Posthoc* moderator analyses will explore the relationship between other study features and effect size and after initial data collection during the full‐text review of included studies.

#### 3.6.1 Planned sensitivity analysis

We do not have any planned sensitivity analyses to specify. During the review process, we do not expect to encounter unusual issues that will be suitable for sensitivity analyses.

#### 3.6.2 Publication selection bias

Publication selection bias will be assessed in three ways. First, analyses will compare the results from published and unpublished reports. Published documents will include peer‐reviewed journal articles, books, and book chapters. All other document forms, such as theses, technical reports, government and agency reports, will be considered unpublished. Second, to model publication bias and small study effects we will use the *meta funnelplot*, *meta bias*, and *meta trimfill* commands in Stata.

#### 3.6.3 Treatment of qualitative research

This review will not include qualitative research.

## ROLES AND RESPONSIBILITIES

- Content: Steven Windisch has extensive background knowledge on terrorism, radicalization, violence, disengagement, and deradicalization. Susann Wiedlitzka has extensive background knowledge on hate crimes, hate speech, racism, and prejudice.
- Systematic review methods: Ajima Olaghere has extensive expertise in statistical analyses. She has coauthored two Campbell Systematic Reviews, one on youth curfews and the other on police‐initiated diversion of low‐risk youth.
- Statistical analysis: Ajima Olaghere and Susann Wiedlitzka have extensive expertise in statistical analyses.
- Information retrieval: Steven Windisch, Ajima Olaghere and Susann Wiedlitzka all have experience performing systematic searches on various topics and retrieving studies and documents for review.

## SOURCES OF SUPPORT

This review is supported by the Horizon 2020 (Grant No.: 699824), DHS Science and Technology Directorate and the Five Research and Development (5RD) Countering Violent Extremism Network.

## DECLARATIONS OF INTEREST

Ajima Olaghere is an editor for the Crime and Justice Coordinating Group within the Campbell Collaboration. She will recuse herself in the review of this protocol and the completed systematic review.

## PRELIMINARY TIMEFRAME

**Table:** 

March 27, 2020	Submit title registration form to Campbell
March—July, 2020	Develop search strategy
May 28, 2020	Title approval received by Campbell
July 9, 2020	Submit protocol for Campbell peer review
July—October, 2020	Develop and implement database and secure storage; revise protocol in response to Campbell review
October—November, 2020	Gather review articles
November—January, 2020	Abstract screening studies for eligibility
January, 2021—February, 2021	Coding, double coding, resolving coding differences; conduct thematic analysis
February—May, 2021	Analyze and interpret data, generate descriptive statistics, conduct meta‐analysis
May—July, 2021	Write draft review
31 July, 2021	Submit draft review for Campbell peer review
16 September, 2021	Revise review and submit to DHS

## PLANS FOR UPDATING THE REVIEW

This review will be updated every 5 years and updating it will be the primary responsibility of Steven Windisch unless all authors agree that another author takes primary responsibility.

## APPENDIX A CODING FORMS FOR QUANTITATIVE STUDIES

**Table:**

**Study Level Coding Form**			
This coding form is for each unique study. Note that a study may be reported in multiple manuscripts (publications, technical reports, etc.). Also, some reports may include the results for distinct studies, such as evaluations in different cities. Our unit‐of‐analysis for the meta‐analysis is an independent study. No two studies should include any of the same participants. If there are multiple publications for the same study, use the most complete study as the primary study ID and all other related studies as cross reference IDs.			
**Identifiers**			
1.	Reference ID	studyid	|__|__|__|__|
2.	Other related references	crossref1	|__|__|__|__|
		corssref2	|__|__|__|__|
		corssref3	|__|__|__|__|
		corssref4	|__|__|__|__|
		corssref5	|__|__|__|__|
3.	Coder's initials	sinitials	|__|__|__|
4.	Creation date (mm/dd/yy)	sdate	|__|__|__|__|__|
5.	Modification date (mm/dd/yy)	sdatem	|__|__|__|__|__|
**General Study Information**			
6.	Publication type	pubtype	|__|
	1. Book 2. Book chapter 3. Journal article (peer reviewed) 4. Journal article (not‐peer reviewed) 5. Thesis‐dissertation 6. Technical report 7. Conference paper 8. Government publication 9. Other (Specify): ______________		
7.	Language type of study	language	|__|
	1. English		
	2. German		
	3. Persian		
	4. Arabic		
8.	Geographic location of study	location	|__|__|__|__|
	1. North America 2. South America 3. Europe 4. Africa 5. Asia 6. Oceania		
9.	Years of data collection		
	Year data collection started	datastart	|__|__|__|__|
	Year data collection ended	dataend	|__|__|__|__|
10.	Intervention type	inttype	|__|
	1. Online only		
	2. Online and offline/mixed approach		
	3. Offline only		
11.	Researcher involvement	rinvolve	|__|
	1. Researcher initiated intervention		
	2. Online platform‐initiated intervention		
	3. Government initiated intervention		
12.	Was this research funded by a grant or external agency	funding	|__|
	0. No		
	1. Yes		
	9. Cannot tell		
**Research Design**			
13.	Unit of assignment to conditions	uoa	|__|
	1. Individual 2. Incident (might include multiple comments) 3. Online platform 4. Online groups 5. Other 9. Cannot tell		
14.	Methodological approach	method	|__|
	1. Qualitative		
	2. Quantitative		
	3. Mixed methods		
15.	How subjects were assigned to condition (this is about assignment not sampling)	assign	|__|
	1. Randomly after matching, yoking, stratification, blocking, etc. 2. Randomly without matching 3. Regression discontinuity (quantitative cutting point defines groups) 4. Wait list control or other such quasi‐random procedures (e.g., alternating cases) 5. Quasi‐experimental, matched individual level 6. Quasi‐experimental, matched group level (e.g., classrooms) 7. Quasi‐experimental, statistical controls for baseline differences 8. Quasi‐experimental, no statistical controls for baseline differences 9. Quasi‐experimental, other 10. Quasi‐experimental, cohort design (historical controls)		
16.	If random assignment or regression discontinuity design:	rndinteg	|__|
	1. Integrity of randomization or other assignment method maintained (no more than a few cases failed to end up in desired group)		
	2. Failures of randomization or assignment occurred		
	3. No information on integrity of assignment process		
17.	[RISK OF BIAS ITEM] Is there any risk of selective outcome reporting bias, that is, is there any evidence that the authors have not reported findings for all variables measured as part of this study?	selectrepb	|__|
	1. Low Risk		
	2. Some Concerns		
	3. High Risk		
18.	Study level coding notes	snotes	
**Comparison Level Coding Form** This coding form is for each treatment/comparison contrast coded from a study. For most studies, you will only code this form once. However, some studies may have two or more treatment conditions or two or more comparison conditions. In the coding below, it is critical to indicate if any of the treatment/comparison contrasts for a study share sample participants. For example, a study might have two distinct treatments but only one comparison group. In this case, these comparisons share sample participants (i.e., the same comparison condition).			
**Identifiers**			
1.	Reference ID	studyid	|__|__|__|__|
2.	Condition ID	compid	|__|__|__|__|
3.	Coder's initials	cinitials	|__|__|__|
4.	Creation date (mm/dd/yy)	cdate	|__|__|__|__|__|
5.	Modification date (mm/dd/yy)	cdatem	|__|__|__|__|__|
6.	Treatment group label	txlabel	|__|__|__|__|__
7.	Control/comparison group label	cglabel	|__|__|__|__|__
**Sample Information**			
8.	Treatment group sample size (at start of study before attrition; −99 if cannot tell)	ctxn	|__|__|__|__|__|
9.	Comparison group sample size (at start of study before attrition; −99 if cannot tell)	ccgn	|__|__|__|__|__|
10.	Mean or median age of sample (−99 if cannot tell)	meanage	|__|__|.__|
11.	Youngest age in sample (−99 if cannot tell)	minage	|__|__|
12.	Oldest age in sample (−99 if cannot tell)	maxage	|__|__|
13.	Sex distribution for this treatment/comparison contrast	sex	|__|
	1. 100% Male 2. 90–99% Male 3. 75–89% Male 4. 26–75% Male 5. 11–25% Male 6. 1–10% Male 7. 0% Male 99. Unknown		
14.	Percent of this condition that is represented by each of the following race/ethnic group (−99 if missing unknown):		
	1. White	white	|__|__|__|.__|
	2. Black/African/Caribbean	black	|__|__|__|.__|
	3. Hispanic (non‐White)	hispanic	|__|__|__|.__|
	4. Asian	asian	|__|__|__|.__|
	5. Mixed/Multiple ethnic groups	mixed	|__|__|__|.__|
	6. Other	raceother	|__|__|__|.__|
**Nature of Treatment Condition**			
15.	Type of intervention	inttype	|__|
	1. Online hate detection only 2. Server shutdowns 3. Deletion of social media accounts 4. Responding to online hate via counter‐narratives 5. Modifying hateful content 6. Countering “fake news” 7. Twitter “fact” check 8. Other (specify): _________		
16.	Content of intervention	intcontent	|__|
	1. Everyday hate 2. Right‐wing extremist content 3. Islamist extremist content 4. Islamist extremist content 99. Cannot tell		
17a.	Location of intervention	intlocate	|__|
	1. Websites 2. Text messaging applications 3. Online and social media platforms		
17b.	If social media, which platform	platform	|__|
	1. Facebook 2. Instagram 3. TikTok 4. WhatsApp 5. Google 6. YouTube 7. Snapchat 8. Twitter 9. 4chan 10. Gab 11. Other (specify): ______________		
18.	Other elements of this condition:	txother	
**Nature of Comparison Condition**			
19.	Type of comparison condition	comptype	|__|
	1. No exposure		
	2. Comparison exposure		
	3. Other		
	[Note: we will add to the list of options as we code studies.]		
20.	Services or sanctions for the comparison condition	compother	
**Comparability of Conditions**			
21.	Were the conditions compared for baseline equivalence on any of the following, either statistically or descriptively? (0 = statistically; 1 = descriptively; 9 = cannot tell)		
	1. Sex	basediff1	|__|
	2. Race	basediff2	|__|
	3. Age	basediff3	|__|
22.	RISK OF BIAS ITEM: Based on the above, is there a risk of selection bias, that is, that the groups were different at baseline?	selectbias	|__|
	1. Low risk		
	2. High risk		
	3. Unclear		
23.	RISK OF BIAS ITEM: Is there a risk of general attrition bias for the primary outcome measure, that is, attrition in excess of 10%?	attrition1	|__|
	1. Low risk		
	2. High risk		
	3. Unclear		
24.	RISK OF BIAS ITEM: Is there a risk of different attrition bias for the primary outcome measure, that is, meaningful differential attrition?	attrition2	|__|
	1. Low Risk		
	2. Some Concerns		
	3. High Risk		
25. Notes about coding this comparison		cnotes	
**Outcome (Dependent Variable) Coding Form**			
Code each eligible outcome or dependent variable using the form below. Note that you should code this only once for a variable that is measured at multiple time points. That is, recidivism measured at 3‐, 6‐, and 9‐months is a single dependent variable. Code the characteristics of the measure using this form and the data for each measurement time point on the effect size forms.			
**Identifiers**			
1.	Reference ID	studyid	|__|__|__|__|
2.	Coder's initials	dvinitials	|__|__|__|
3.	Creation date (mm/dd/yy)	dvdate	|__|__|__|__|__|
4.	Modification date (mm/dd/yy)	dvdatem	|__|__|__|__|__|
5.	Outcome ID	dvid	|__|__|__|__|
6.	Dependent variable label	dvlabel	|__|__|__|__|
**Characteristics of Variable**			
7.	Elements reported in this outcome measure irrespective of the type of incident and reporting source (check best one):	dvelem	|__|__|__|
	1. Global dichotomy or polychotomy (e.g., created, or consumed cyberhate, extremist content or non‐extremist content = yes/no)		
	2. Summed dichotomous (e.g., sum of “yes/no” on list of specific behaviors)		
	3. Frequency or rate, (count of incident; incidents per 1000 persons)		
	4. Severity (seriousness rating or index), see this often with self‐report measures		
	5. Event timing (e.g., days without content creation; time since last post, log on, video watch)		
	6. Proportion or amount of time on extremist website, etc.		
	7. Rating of amount of delinquency, severity, change, etc. This is similar to frequency but in rating form. (e.g., How often you did “x” behavior)		
	8. More than one of above elements combined in composite measure		
	9. Other		
	99. Cannot tell		
8.	Type of behavior represented by this measure (what's counted, irrespective of source of information and authors' label or description of the measure) check best one:	dvtype	|__|__|__|
	1. Content creation (e.g., production and authorship of original content such as making videos, writing blog posts, or uploading content)		
	2. Transmission of hate speech (e.g., racist, homophobic, anti‐Semitic), not specifically restricted to extremist acts		
	3. Consumption of cyberhate (e.g., watch videos, visit social media platforms, or read blogs without making accounts from self or observer's report)		
	4. Collecting extremist content (e.g., organize links and content for either their personal use or to disseminate information to others who are active online		
	5. Critics (e.g., comment on social media posts, submit reviews, and rate content)		
	6. Joiners (e.g., those who maintain accounts but do not comment or post publicly available content)		
	7. Other		
	99. Cannot tell		
9.	RISK OF BIAS ITEM: Person providing outcome data knows which condition the participant is in (i.e., is there a potential bias from the lack of blinding of the assessor?)	dvbias	|__|
	1. Low Risk		
	2. Some Concerns		
	3. High Risk		
10.	Notes regarding this outcome measure	dvnotes	

**Effect Size Coding Form**			
Code all effect sizes of interest using the form below, coding each effect size separately (i.e., with a different copy of the form or record in the database). Indicate the study ID, comparison ID, and dependent variable ID. Give each effect size within a study a unique ID (i.e., 1, 2, 3…).			
There are several ways to compute effect sizes using the different tabs. ONLY USE ONE METHOD per effect size. If you have the raw means and also a regression coefficient for the same outcome from a model that adjusts for baseline differences, these are two different effect sizes. The different effect size computation methods are:			
1. Means and standard deviations 2. Means and standard errors 3. Frequency of failures in each condition 4. Proportion of failures in each condition 5. Logistic regression coefficient for treatment effect dummy code 6. OLS unstandardized regression coefficient 7. OLS standardized regression coefficient 8. Independent samples *t* test 9. Chi‐square test (2 by 2, df = 1) 10. Point‐biserial correlation coefficient 11. Phi correlation coefficient 12. Hand computation (e.g., using the online effect size calculator)			
**Identifiers**			
1.	Reference ID	studyid	|__|__|__|__|
2.	Coder initials	esinitials	|__|__|__|
3.	Creation date	esdate	|__|__|__|__|__|
4.	Modification date	esdatem	|__|__|__|__|__|
5.	Comparison ID	compid	|__|__|__|__|
6.	Outcome ID	dvid	|__|__|__|__|
7.	Effect Size ID	esid	|__|__|__|__|
**Effect Size Information**			
8.	Direction of effect	esdirect	|__|
	1 = favors treatment		
	2 = favors control		
	3 = neither, exactly equal		
	99 = cannot tell		
9.	Type of effect size (i.e., baseline differences, first post treatment outcome measure, or a follow‐up measure)	estype	|__|
	1 = baseline (pretest)		
	2 = posttest		
	3 = follow‐up		
10.	Effect reported as statistically significant by authors	essig	|__|
	0 = no		
	1 = yes		
	99 = cannot tell		
11.	Timing of measurement (months captured by the measure from the point of assignment to conditions or diversion/formal processing; if reported in months, divide by 4.3; 8888 if not applicable; 9999 if missing)		
	Mean	estime1	|__|__|__|__|
	Minimum	estime2	|__|__|__|__|
	Maximum	estime3	|__|__|__|__|
**Effect Size Data**			
12.	Treatment group sample size for this effect size	estxn	|__|__|__|__|
13.	Comparison group sample size for this effect size	escgn	|__|__|__|__|
	***Scaled outcome data***		
14.	Mean treatment group	esmtx	|__|__|__|__|.__|__|
15.	Mean comparison group	esmcg	|__|__|__|__|.__|__|
16.	Are the above means adjusted for baseline differences? 0 = no; 1 = yes; 99 = cannot tell)	esmadj	|__|
17.	Standard deviation treatment group	essdtx	|__|__|__|__|.__|__|
18.	Standard deviation comparison group	essdcg	|__|__|__|__|.__|__|
19.	Standard error treatment group	essetx	|__|__|__|__|.__|__|
20.	Standard error comparison group	essecg	|__|__|__|__|.__|__|
	***Dichotomous outcome data***		
21.	Treatment group number successful	Estxn	|__|__|__|__|
22.	Comparison group number successful	Escgn	|__|__|__|__|
23.	Treatment group number failures	estxnf	|__|__|__|__|
24.	Comparison group number failures	escgnf	|__|__|__|__|
25.	Treatment group proportion of successes (only code this if raw frequencies are not available)	estxpf	|__|.__|__|__|__|__|
26.	Comparison group proportion of successes (only code this if raw frequencies are not available)	escgpf	|__|.__|__|__|__|__|
27.	Are the above frequencies or proportions adjusted for baseline differences? (1 = yes; 0 = no; 9 = cannot tell)	espadj	|__|
	***Logistic regression***		
28.	Logistic regression coefficient (for treatment effect dummy)	eslgor	|__|.__|__|__|__|__|
29.	Standard error for logistic regression coefficient	esselgor	|__|.__|__|__|__|__|
30.	*t* test or *z* test for logistic regression coefficient	esolst	|__|.__|__|__|__|__|
31.	Odds ratio for treatment effect dummy (optional)	esor	|__|__|__|.__|__|__|
	***OLS regression***		
32.	Unstandardized regression coefficient	esolsb	|__|.__|__|__|__|__|
33.	Standard regression coefficient	esolsbeta	|__|.__|__|__|__|__|
34.	Standard error of regression coefficient	esolsse	|__|.__|__|__|__|__|
35.	Standard deviation for dependent variable	essd	|__|.__|__|__|__|__|
	***Other possible effect size data***		
36.	*t* test (comparing two‐sample means; not the *t* from a regression model)	est	|__|__|__|__|.__|__|
37.	*p* value from a *t* test (comparing two‐sample means; not the *t* from a regression model)	espfromt	|__|.__|__|__|__|__|
38.	Correlation coefficient point‐biserial (treatment versus comparison correlated with scaled variable)	esrpb	|__|.__|__|__|__|__|
39.	Correlation coefficient phi (treatment versus comparison correlated with a dichotomous variable)	esrphi	|__|.__|__|__|__|__|
40.	Chi‐square (treatment versus comparison correlated with a dichotomous variable, *df* must equal 1)	eschisq	|__|__|__|__|.__|__|
	***Effect size computed by hand (e.g., using online calculator)***		
41.	Standardized mean difference effect size computed by hand (*d*‐type)	eshand	|__|__|.__|__|__|__|
42.	Variance for standardized mean different effect size computed by hand	eshandv	|__|__|.__|__|__|__|
43.	Computed effect size	escalc	|__|__|.__|__|__|__|
44.	Computed effect size standard error	escalcse	|__|__|.__|__|__|__|
	***Effect size coding notes***		
45.	Page number where effect size data found	espage	|__|__|__|__|__|__|
46.	Notes about this effect size	esnotes	
